# Gene Network Mechanism of Zhilong Huoxue Tongyu Capsule in Treating Cerebral Ischemia–Reperfusion

**DOI:** 10.3389/fphar.2022.912392

**Published:** 2022-07-07

**Authors:** Na Li, Jie Sun, Ji-Lin Chen, Xue Bai, Ting-Hua Wang

**Affiliations:** ^1^ Liaoning Key Laboratory of Diabetic Cognitive and Perceptive Dysfunction, Jinzhou Medical University, Jinzhou, China; ^2^ Department of Neurosurgery, West China Hospital, Sichuan University, Chengdu, China; ^3^ Department of Encephalopathy, Affiliated Hospital of Traditional Chinese Medicine, Southwest Medical University, Luzhou, China

**Keywords:** ZhiLong HuoXue TongYu capsule, cerebral ischemia–reperfusion, network pharmacology, molecular docking, gene network analysis

## Abstract

**Objective:** To investigate the effect of Zhilong Huoxue Tongyu capsule (ZLH) in the treatment of cerebral ischemia–reperfusion injury and determine the underlying molecular network mechanism.

**Methods:** The treatment effect of Zhilong Huoxue Tongyu capsule (ZLH) was evaluated for cerebral ischemia–reperfusion injury in middle cerebral artery occlusion (MACO) rat, and the underlying molecular network mechanism was explored by using molecular network analysis based on network pharmacology, bioinformatics including protein–protein interaction (PPI) network, Gene Ontology (GO), and Kyoto Encyclopedia of Genes and Genomes (KEGG), as well as molecular docking.

**Results:** The neurological function of rats in the ZLH group was significantly improved compared to those in the NS group (*p* = 0.000), confirming the positive effect of ZLH for the treatment of brain ischemia. There were 126 intersecting genes screened in ischemia–reperfusion cerebrum that are associated with several important biological processes, such as lipopolysaccharide, and the most important cell component, such as raft, as well as the most important molecular function pointed as cytokine receptor binding. The most important KEGG signaling pathway was the AGE-RAGE signaling pathway in diabetic complications. Moreover, according to the STRING interaction in the PPI network, 10 hub genes including MAPK14, FOS, MAPK1, JUN, MYC, RELA, ESR1, STAT1, AKT1, and IL6 were selected and exhibited in Cytoscape and molecular docking. Lastly, the relation between PPI, GO, and KEGG was analyzed. These findings indicated that multiple hub network genes have been involved in behavior improvement in cerebral ischemia–reperfusion rats subjected to ZLH treatment.

**Conclusion:** Zhilong Huoxue Tongyu capsule improves cerebral ischemia–reperfusion and is associated with multiple network gene expressions.

## Introduction

Stroke, one of the three most common causes of death and disability, is increasing with the high-pressure life. About 70% of strokes are derived from ischemic, and the rest are intracerebral or subarachnoid hemorrhages (Sveinsson et al., 2014a). After stroke, patients suffered from various neurological disorders that prevent them from taking care of themselves, resulting in a serious impact on their quality of life, psychologically, as well as socially and financially, for both the patients and their families (Sveinsson et al., 2014b). Cell death may occur within minutes after cerebral ischemia attack, and thrombolytic therapy can be used to restore blood flow and reduce the morbidity and mortality of patients. It has been known that microcirculatory dysfunction and organ damage after cerebral ischemia–reperfusion, as a complex pathological process, play a crucial role in the process of cerebral ischemia–reperfusion, and the blood supply in the organ is first restricted, while the perfusion could restore and reoxygenate (Han et al., 2017). Cerebral ischemia–reperfusion causing the ischemic penumbra cell death may lead to additional injury in the bordering locations of the infarct core, which needs to develop several new methods so as to improve treatment effect.

Zhilong Huoxue Tongyu Capsule (ZLH) (Sichuan Medicine Z20070528) is an available traditional Chinese medicine (TCM) that could be utilized for the treatment of cerebral stroke (Liang et al., 2021; Liu et al., 2022). As a complex preparation, ZLH, consisting of *Astragalus*, Cassia twig, leech, *Panax notoginseng*, and chuanxiong, is effective in dispelling wind and dampness and relieving pain for vascular headache and migraine. Among of them, *Astragalus* can hold the surface for qi and fold sweat; cassia twig can generate muscle, warm the meridians, and pass through Yang Qi; leech can precipitate and break blood clots (Liu et al., 2021). However, the molecular network mechanism of ZLH in improving brain damage is largely unknown.

Network pharmacology is based on core target analysis, network interaction relationship analysis, and description of key biological processes and signaling pathways to explain the disease–target-drug co-association module, which could exhibit the network regulatory relationship between drugs and diseases, so as to uncover the mechanism of pharmaceutical agents (Li and Zhang, 2013; Zhao et al., 2019; Luo et al., 2020), especially molecular docking that reveals the relationship between the molecular structural formula and drug, and it may help us to find the hub gene to predict ligand–target interactions with drug components (Pinzi and Rastelli 2019; Song et al., 2020). All of these techniques could be used to explain the molecular network mechanism in diseases and drug addition.

In this study, we investigated the protective effect and network mechanism of ZLH against cerebral ischemia–reperfusion, by gene network analysis, molecular docking, and animal experiment.

## Materials and Methods

### Animals and Grouping

A total of 33 SD rats were provided by the Department of Experimental Animals, Kunming Medical University (No. SCXK (Yunnan) K2020-0004); the animal ethics code is KMMU20220854, and the rats were randomly grouped into the sham group (*n* = 12), NS group (*n* = 10), and ZLH group (*n* = 11). After 8 h of fasting, the rats were deeply anesthetized with 3% sodium pentobarbital and performed the right common carotid artery occlusion in rats, by inserting a wire into the internal carotid artery. Then, the wound was sutured till the embolization was removed after 1 h (Liu Haixiao et al., 2019).

### Drug Therapy

A total of 40 capsules of ZLH were dissolved in 100 ml of saline (concentration of 16 g/ml), and 2 ml/day was intragastrically administered to each rat in the ZLH group. The rats in the NS group were given 2 ml/day of saline, and the sham group was fed and watered as usual. All rats in each group were observed until sacrificed at day 5.

### Zea-Longa Scores

All rats were assessed 24 h after awakening from anesthesia by using Zea-Longa scores. The scoring criteria were as follows: 1) no signs of nerve damage, 0 points; 2) inability to fully extend the contralateral front paw, 1 point; 3) rotate to the opposite side while crawling, 2 points; 4) falling to the opposite side while standing or crawling, 3 points; 5) inability to walk on their own and loss of consciousness, 4 points. The higher the score indicates the more severe the behavioral disorder of the animal.

### Searching for Drug Components and Targets

The components and related target proteins of *Astragalus*, Cassia twig, leech, *Panax notoginseng*, and chuanxiong in ZLH were extracted by TCMSP (https://old.tcmsp-e.com/tcmsp.php), according to the condition of oral bioavailability (OB) that was set at > 20 and drug similarity (DL) was at >0.15. After retrieving the active ingredient of the drug, the name of the target protein corresponding to the active ingredient is checked in “related targets” according to the molarity of the active ingredient, and the name of the gene corresponding to the target protein is correspondingly searched in UniProt.

### Searching for Gene Clusters of Cerebral Ischemia–Reperfusion

After entering the keyword “cerebral ischemia-reperfusion” in GeneCards (https://www.genecards.org/), we downloaded a list of genes related to cerebral ischemia–reperfusion.

### Venny Intersection Diagram

The genes involved in cerebral ischemia–reperfusion were screened, and the Venny intersection map with ZLH was constructed (Venny 2.1.0, https://bioinfogp.cnb.csic.es/tools/venny/index.html). We input the gene name using “BI” in List1 and “ZL” in List2 and then constructed the Venny intersection map.

### Gene Ontology Enrichment and Kyoto Encyclopedia of Genes and Genomes Pathway Analysis

The R software (version 4.1.0) was used to perform the GO enrichment and KEGG pathway analysis of the intersection obtained in Venny. In the Java environment, after the computer was first installed with the activePerl.exe file, org.Hs.eg.db, DOSE, clusterProfiler and pathView Enrichplot, ” we then used the scripts of SymolID, GO, and KEGG file packages to analyze the gene network, which includes the biological processes (BP), cellular components (CC), and molecular functions (MF), and the KEGG signaling pathway that can be obtained.

### Protein–Protein Interaction Network

PPI network analysis (https://cn.string-db.org/) was performed using the intersection derived from the analysis in Venny 2.1.0. The results were then obtained by selecting multiple proteins and entering the intersecting genes in the “list of name” and selecting “*Homo sapiens*” for analysis.

### Screening of Hub Genes

The interaction result obtained from PPI analysis was imported into Cytoscape 3.8.2 to screen hub genes, and 10 hub genes were screened based on degree values.

### Molecular Docking

The screened hub genes were input into PDB, and the corresponding protein structures were downloaded; then, the corresponding gene drugs were entered into PubChem, and their 2D structures were downloaded; then, the SDF format of the receptors was converted to the MOL2 format using Open Babel. Subsequently, the ligand and receptor are then separated by dehydration using PyMOL software, with the target protein of the gene as the receptor and the corresponding drug as the ligand. Next, molecular docking was performed in AutoDock Vina to complete the docking and analysis, and the docking results were exported in the PDBQT format and converted from the PDBQT format to PDB format using Open Babel. Finally, PyMOL software was used to adjust the color of the large protein, process the ligands and residues, and calculate the length of hydrogen bonds. Lastly, the ligand and the overall picture are exported.

### Cytoscape Diagram of Disease–Drug Active Component–Key Target–KEGG Pathway

All tables were imported into Cytoscape and divided into five types, including 1) type 1, drug mole number and corresponding genes; 2) type 2, disease name and disease gene; 3) type 3, KEGG signaling pathways and corresponding genes; 4) type 4, drug name and mole number; 5) type 5, KEGG signaling pathways. The disease–drug active component–key target–KEGG pathway diagram was constructed rationally by changing its shape, color, and position.

## Results

### Gene Cluster for ZLH and Cerebral Ischemia–Reperfusion

A total of 199 genes of components and related target proteins for ZLH were examined (Table 1), and 1,493 genes for cerebral ischemia–reperfusion were screened out (Table 2).

**TABLE 1:** Gene list of ZLH.

PGR	PRSS3	GSK3B	OPRM1	ATP5F1B	HMOX1	SLC2A4
NOS2	PYGM	CCNA2	KCNH2	ND6	CYP3A4	NR1I3
PTGS1	CHRM3	AKR1B1	CHRM5	HSD3B2	CYP1A2	INSR
AR	CHRM1	F7	CHRM4	HSD3B1	CYP1A1	DIO1
SCN5A	CHRM2	ACHE	OPRD1	IKBKB	ICAM1	PPP3CA
PTGS2	ADRA1B	MAOB	ADRA1A	AKT1	SELE	GSTM1
ESR2	GABRA1	RELA	SLC6A3	BCL2	VCAM1	GSTM2
CHEK1	GRIA2	NCF1	SLC6A4	BAX	NR1I2	AKR1C3
PRSS1	ADH1B	OLR1	RXRB	TNFSF15	CYP1B1	SLPI
NCOA2	RXRA	ADRB1	KDR	AHSA1	ALOX5	MMP3
NR3C2	SLC6A2	HTR3A	MET	CASP3	HAS2	EGFR
NCOA1	ESR1	ADRA2C	PKIA	MAPK8	GSTP1	VEGFA
ADH1C	PPARG	ADRB2	JUN	MMP1	AHR	CCND1
LYZ	MAPK14	ADRA1D	IL4	STAT1	PSMD3	BCL2L1
CCNB1	RASSF1	CD40LG	CHRNA2	SLC2A1	PRKCD	ABCG2
PLAT	E2F1	IRF1	MAP2	LTA4H	CSF2	NFE2L2
THBD	E2F2	ERBB3	CAT	FASN	ACTA2	NQO1
SERPINE1	ACPP	PON1	DGAT2	KLF7	BTK	PARP1
COL1A1	CTSD	PCOLCE	MTTP	NFKBIA	FLT4	COL3A1
FOS	RB1	POR	ERBB2	GJA1	DUOX2	CXCL11
CDKN1A	IL6	ODC1	ACACA	IL1B	NOS3	CXCL2
EIF6	TP63	CASP8	CAV1	CCL2	HSPB1	DCAF5
CASP9	ELK1	TOP1	MYC	PTGER3	SULT1E1	CHEK2
PLAU	APOB	RAF1	F3	CXCL8	MGAM	CLDN4
MMP2	FLT1	SOD1	IFNG	PRKCB	IL2	PPARA
MMP9	PRKCE	PRKCA	IL1A	BIRC5	IGFBP3	PPARD
MAPK1	CXCL10	HIF1A	MPO	NPEPPS	IGF2	HSF1
IL10	CHUK	RUNX1T1	TOP2A	HK2	RASA1	CRP
EGF	SPP1	RUNX2				

**TABLE 2:** Partial gene list of cerebral ischemia–reperfusion.

APP	VEGFA	COL4A2	THBD	PTGS2	NPPA	ADAMTS13
KRIT1	PTEN	BCL2	CAT	BAX	TEK	SLC2A1
CST3	IL10	BDNF	VWF	KNG1	TSC2	APOH
F2	MEF2C	XDH	HSPA4	SERPINI1	CASP9	PROC
IL6	CASP3	NOS1	ALB	CXCL12	GJA1	EGR1
TNF	PLAT	CCL2	MB	GRIK2	FLT1	IFNG
NOS3	SMARCA4	CBS	GRIN2B	GP1BA	PON1	VLDLR
COL4A1	MAPT	SLC1A2	TGFB2	PIK3C2A	HSPA5	INS
ACE	GFAP	HMOX1	MMP2	GSR	AKT1	AGT
F5	EDN1	MMP9	IGF1	CD36	SHH	GRIN2A
NOS2	PSEN1	SELE	ENO2	CDK5	SNCA	ITGB3
ENG	SERPINC1	EPO	NPPB	TGFB1	GRIN1	HMGB1
SOD1	TLR4	SELP	F3	KCNJ5	AGTR1	CALCA
MTHFR	HIF1A	S100B	IL1RN	ADM	MAPK14	FGF2
ICAM1	PRNP	SOD2	ACTA2	ODC1	IL18	IL4
PIK3CA	CTNNB1	JAK2	SETD2	CYCS	YRDC	ADORA2A
MPO	IL1B	MIR21	SERPINA3	PDGFB	MTOR	MAPK1
GAD1	COL3A1	SERPINE1	PLA2G6	NFE2L2	SMARCAL1	LONP1
TP53	CRP	CXCL8	KDR	MAP2	PLG	CASP1
F13A1	TXN	MBL2	PECAM1	TGFBR2	MME	COX5A
ATP1A2	JUN	CREB1	ATM	TSPO	NGB	ARID1B
AIFM1	CTLA4	NTRK2	ANGPT2	HTRA2	HTR2A	LPL
ANGPT1	CP	CTSD	TIMP1	SLC9A1	THBS1	EDNRA
APOB	EGF	ITPR1	LOX	GPT	TGIF1	EDNRB
PARP1	ADRB2	FGB	FLNA	SLC6A4	GDNF	SMAD4
FAS	IDH1	HGF	SLC8A1	CSF1R	LAMB1	RELA
CSF3	LDLR	TLR2	ERCC2	PF4	HSPG2	TGFBR1
HSPA1A	APOA1	FOS	ANXA5	PTGS1	MT-CO1	ADA
AQP4	HSPB1	HSPA8	ITGAM	PRKCE	RPS27A	MIR155
SLC6A3	ALOX5	MIR146A	CTSB	PRKAA2	ADIPOQ	ACTB
SPTAN1	SLC17A5	PPARG	PLAU	TOMM40	IL1A	DRD2
G6PD	PROCR	GLUL	NES	MIR34A	NCF1	TIMP3
VCAM1	THPO	CD40LG	SLC12A2	PDP1	CDKN3	MIR145
LTA	PSAP	DLG4	SMAD2	REN	SH2B3	PIK3CG
MPL	FGA	EPRS1	EGFR	PTK2B	GAPDH	CDKN2A
CR1	CLU	NPY	LEP	HP	VCP	PPARA
ACHE	NGF	SLC1A1	NOTCH1	SELL	AOC3	NLRP3
ADORA1	MAPK8	MIR210	BAD	ADORA3	SST	CDON
CALR	TIMP2	LMNA	STAT1	TNFRSF1A	GSS	TNNI3

### Intersection Genes Between ZLH and Cerebral Ischemia–Reperfusion

**FIGURE 1:**
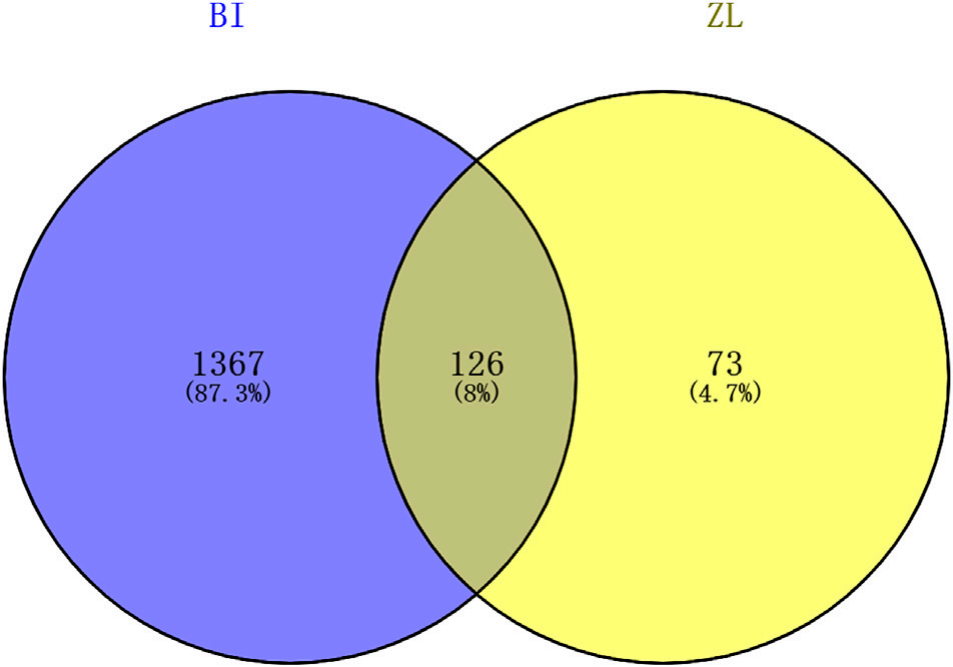
Venny intersection diagram between cerebral ischemia–reperfusion and ZLH.

A total of 199 genes for ZLH and 1,493 genes for cerebral ischemia–reperfusion were crossed in Venny 2.1.0, and 126 intersecting genes were obtained (Figure 1; Table 3).

**TABLE 3:** Intersection genes for ZLH and cerebral ischemia–reperfusion.

IL6	CXCL8	IL4	IL2	IGF2	CHUK	SLPI
NOS3	THBD	MAPK1	BCL2L1	CSF2	ABCG2	CHRM3
NOS2	CAT	APOB	ESR1	GSK3B	RUNX2	FASN
SOD1	MMP2	PARP1	SLC6A4	MYC	OPRD1	BTK
ICAM1	F3	SLC6A3	PTGS1	CAV1	SLC6A2	POR
MPO	ACTA2	VCAM1	PRKCE	MAOB	PRKCA	CHEK1
VEGFA	KDR	ACHE	IL1A	IRF1	GSTM1	HMOX1
IL10	PTGS2	JUN	NCF1	PRKCD	CXCL11	MMP9
CASP3	BAX	EGF	RELA	CCND1	CHEK2	SELE
PLAT	ODC1	ADRB2	PPARA	CYP1B1	NFKBIA	SERPINE1
HIF1A	NFE2L2	HSPB1	IGFBP3	PRKCB	CHRM2	AKT1
IL1B	MAP2	ALOX5	SPP1	OPRM1	BIRC5	MAPK14
COL3A1	CASP9	MAPK8	ADRB1	HTR3A	PTGER3	SLC2A1
CRP	GJA1	CTSD	OLR1	RAF1	MGAM	IFNG
BCL2	FLT1	FOS	SCN5A	MMP1	KCNH2	CD40LG
CCL2	PON1	PPARG	CXCL2	GSTP1	PPARD	PLAU
CXCL10	NR3C2	HK2	AKR1B1	NQO1	E2F1	EGFR
COL1A1	INSR	ACACA	CASP8	CDKN1A	HSF1	STAT1

### GO Analysis

**FIGURE 2:**
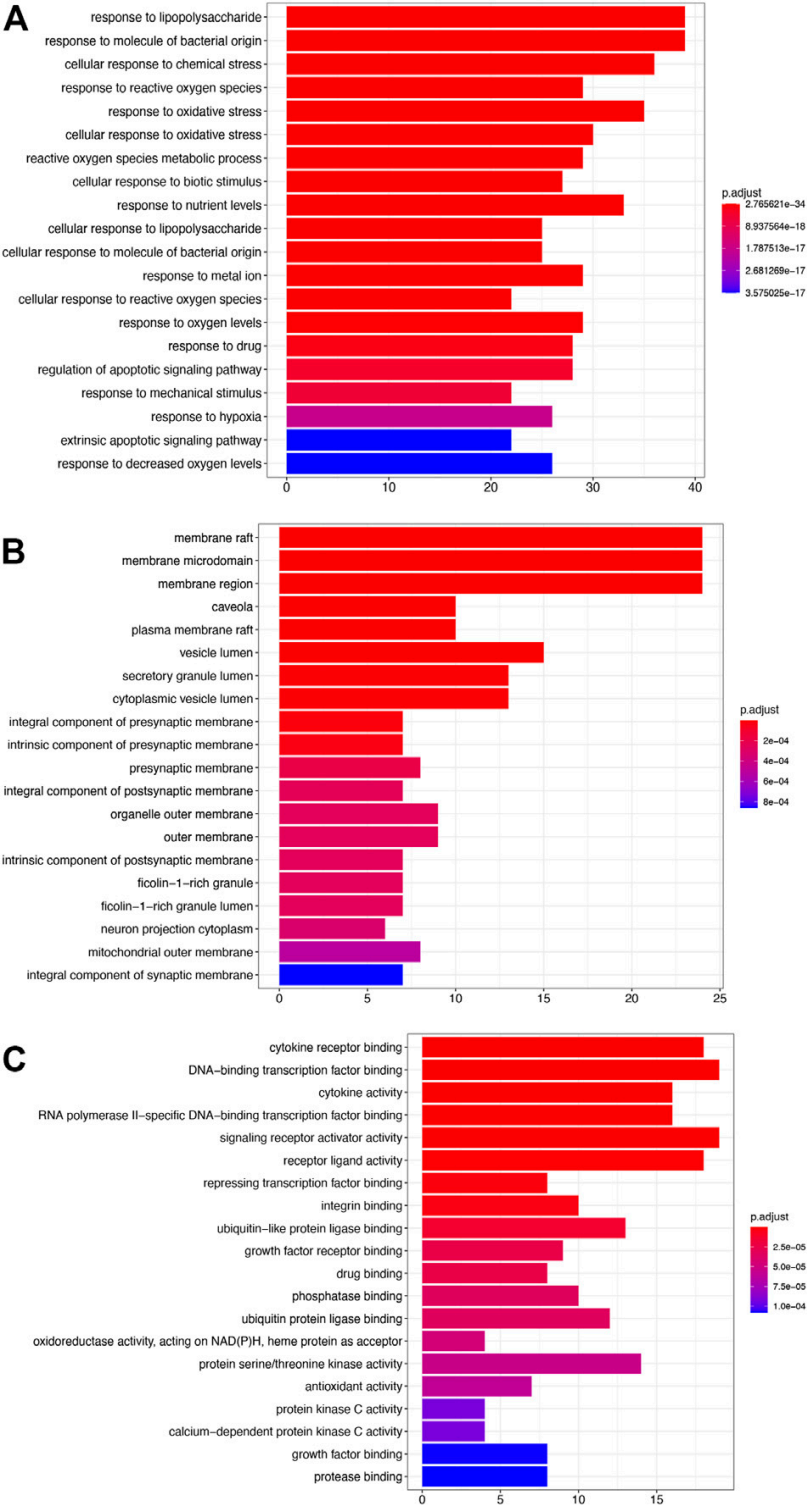
GO analysis. **(A)** Biological processes (BP). **(B)** Cellular components (CC). **(C)** Molecular functions (MF).

The results of enrichment analysis showed that the first 20 pathways of biological process (BP), cellular component (CC), and molecular function (MF) were analyzed. The most important biological process was response to lipopolysaccharide; the main component of lipopolysaccharide was the outer membrane of Gram-negative bacteria, leading to the immune reaction. It plays an early warning role in inflammatory response after cerebral ischemia–reperfusion. The most important cell group is *raft*, which plays a regulatory role in the area around cerebral infarction. The most important molecular function is cytokine receptor binding, which activates intracellular enzymes to regulate gene expression (Figure 2).

### KEGG Signaling Pathways

**FIGURE 3:**
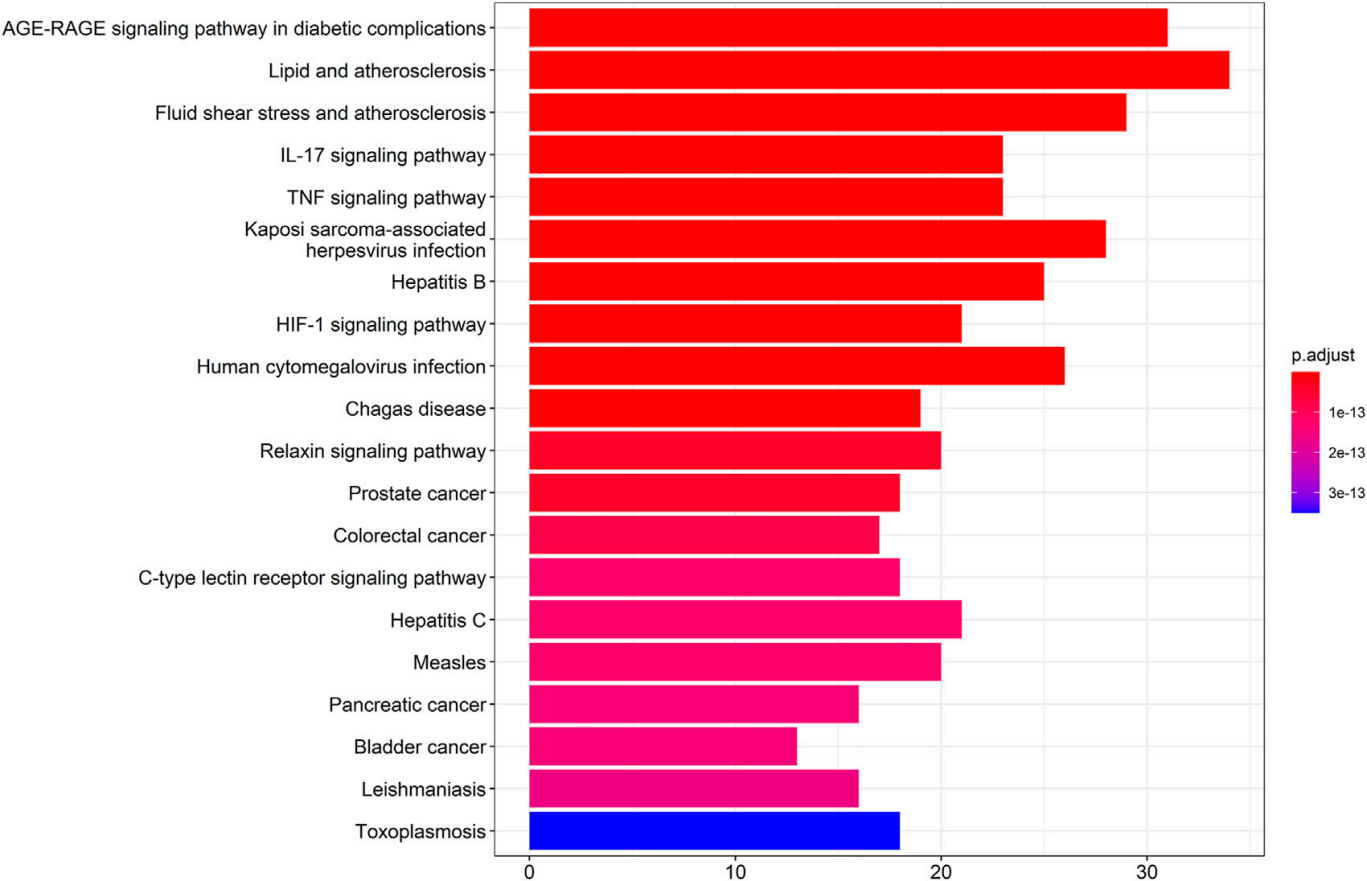
KEGG signaling pathways.

The results showed that the top 10 KEGG signaling pathways were as follows: AGE-RAGE signaling pathway in diabetic complications, lipid and atherosclerosis, fluid shear stress and atherosclerosis, IL-17 signaling pathway, TNF signaling pathway, Kaposi sarcoma-associated herpesvirus infection, hepatitis B, HIF-1 signaling pathway, human cytomegalovirus infection, and Chagas disease (Figure 3).

### PPI Network Analysis and Hub Genes Screening

#### PPI Network Analysis

**FIGURE 4:**
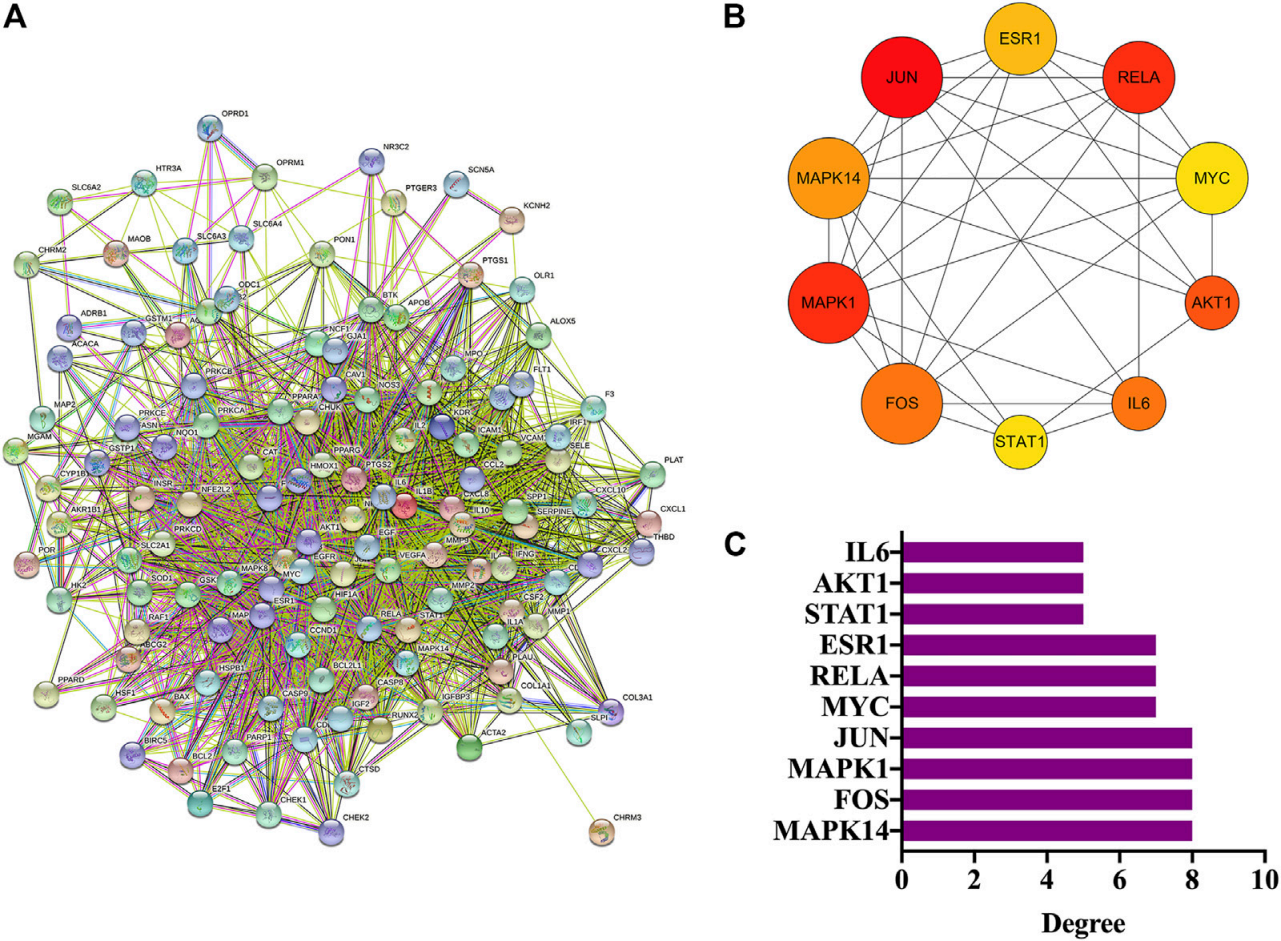
PPI network analysis and hub genes. **(A)** PPI network analysis. **(B)** Screening of hub genes. **(C)** Degree value of hub genes.

In total, 126 intersection genes were obtained from Venny 2.1.0, shown in Figure 4A and Table 4). The 10 pairs of genes that interacted most closely according to COMBINed_score in descending order were: FLT1: VEGFA, AKT1: NOS3, CAV1: EGFR, CAV1: NOS3, CCND1: CDKN1A, CCND1: ESR1, CHUK: NFKBIA, EGF: EGFR, ESR1: JUN, and FOS: JUN.

**TABLE 4:** | PPI network analysis.

#Node1	Node2	Co-expression	Experimentally_determined_interaction	Database_annotated	Automated_text mining	Combined_score
FLT1	VEGFA	0.062	0.978	0.9	0.991	0.999
AKT1	NOS3	0.049	0.879	0.9	0.988	0.999
CAV1	EGFR	0.335	0.881	0.9	0.988	0.999
CAV1	NOS3	0	0.835	0.9	0.989	0.999
CCND1	CDKN1A	0.085	0.983	0.9	0.99	0.999
CCND1	ESR1	0	0.867	0.9	0.987	0.999
CHUK	NFKBIA	0.049	0.994	0.9	0.935	0.999
EGF	EGFR	0.16	0.982	0.9	0.991	0.999
ESR1	JUN	0	0.684	0.9	0.988	0.999
FOS	JUN	0.656	0.985	0.9	0.996	0.999

#### Screening of Hub Genes

The top 10 genes were sorted by the degree value, and the circle from the largest to smallest represented the decreasing degree value. As shown, the top 10 hub genes were MAPK14, FOS, MAPK1, JUN, MYC, RELA, ESR1, STAT1, AKT1, and IL6 (Figures 4B,C).

### Results of Molecular Docking

**FIGURE 5:**
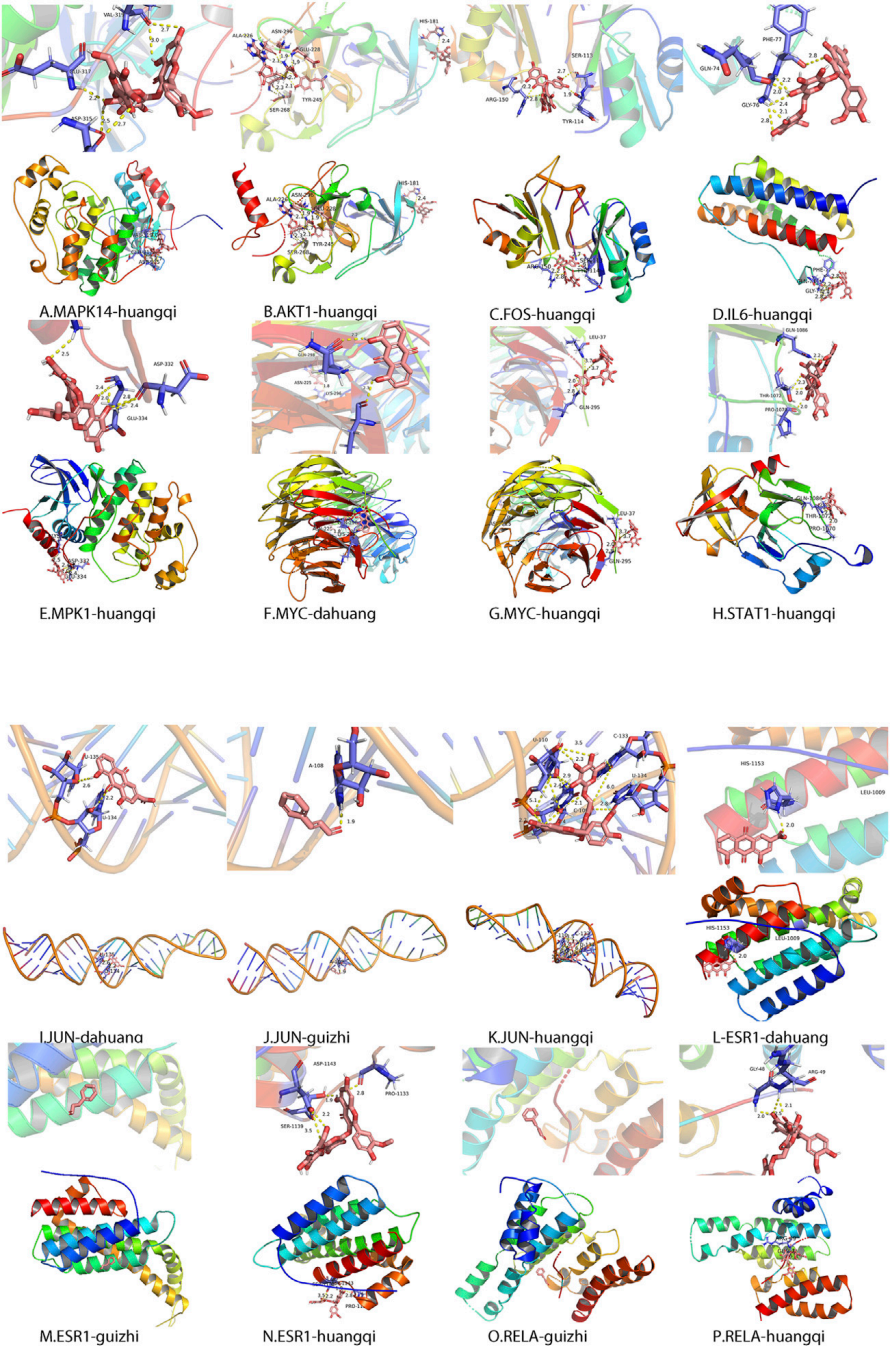
Molecular docking of related protein molecules of ZLH. **(A)** MAPK14–Huangqi. **(B)** AKT1–Huangqi. **(C)** FOS–Huangqi. **(D)** IL-6–Huangqi. **(E)** MAPK1–Huangqi. **(F)** MYC–Dahuang. **(G)** MYC–Huangqi. **(H)** STAT1–Huangqi. **(I)** JUN–Dahuang. **(J)** JUN–Guizhi. **(K)** Jun–Huangqi. **(L)** ESR1–Dahuang. **(M)** ESR1–Guizhi. **(N)**ESR1–Huangqi. **(O)** RELA–Guizhi. **(P)** RELA–Huangqi.

Based on the 10 target proteins screened in Cytoscape, molecular docking was carried out with ZLH. The crystal structures of the target proteins were downloaded from the PDB database, formatted by Open Babel, and molecular docking of the target proteins to the ZLH was performed in AutoDock Vina. Finally, PyMOL software was used to process and adjust the color of the large protein, process the ligands and residues, calculate the length of hydrogen bonds, and export the ligands and the overall picture (Figure 5).

### Cytoscape Map

**FIGURE 6:**
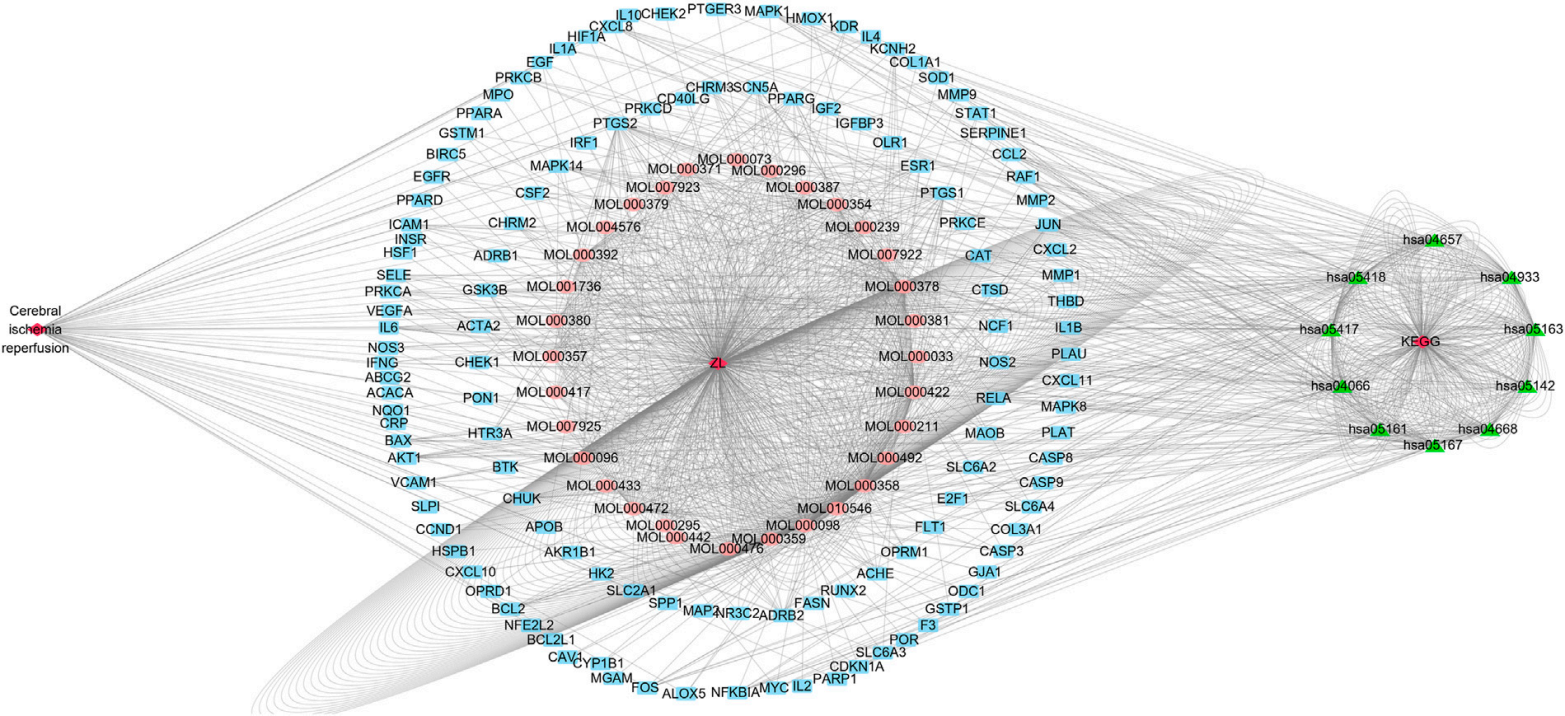
Cytoscape diagram for cerebral ischemia–reperfusion, ZLH administration, and KEGG signaling pathways. The left exhibited cerebral ischemia–reperfusion disease and the middle was the MOL number from ZLH and associated 44 active components, whereas the outer blue circle was the common gene of cerebral ischemia–reperfusion and ZLH. Lastly, the first 10 KEGG signaling pathways were put on the right side.

The disease–drug active component–key target–KEGG signaling pathway diagram was constructed by importing the 44 active ingredients corresponding to ZLH, 126 common cross-targets of cerebral ischemia–reperfusion and ZLH, and the first 10 pathways of KEGG into Cytoscape software. As a result, we constructed the network interaction among all related targets, in which, the closely related pathways were linked in the figure to illustrate the regulatory role of ZLH on cerebral ischemia–reperfusion (Figure 6).

### The Relation of Hub Genes With PPI, GO, and KEGG Pathway

To know the relation and link of hub genes with PPI, GO, and KEGG pathway, we compared the position of hub genes in the GO and KEGG pathway. We found that almost all hub gens were involved in BP and selected signal pathways, but only MAPK1 or Mapk14 were involved in CC. These pointed out that 10 hub genes selected from PPI are most importantly related to the findings of GO analysis and signal pathway. In addition, the molecular hub genes like MAPK14, FOS, MAPK1, and JUN may simultaneously locate in different signaling pathways involved in the AGE-RAGE signaling pathway in diabetic complications, lipid and atherosclerosis, fluid shear stress and atherosclerosis, IL-17 signaling pathway, TNF signaling pathway, Kaposi sarcoma-associated herpesvirus infection, hepatitis B, HIF-1 signaling pathway, human cytomegalovirus infection, and Chagas disease, which means that some co-expression genes scattered in different signaling pathways. In turn, the same signaling pathway like the IL-17 signaling pathway includes different genes including MAPK14, FOS, MAPK1, JUN, RELA, and AKT1, simultaneously (Table 5).

**TABLE 5:** Hub genes enriched in GO and KEGG pathway table.

BP	Description	Gene ID							
BP	Response to lipopolysaccharide	MAPK14	FOS	MAPK1	RELA	AKT1	IL6		
BP	Response to molecule of bacterial origin	MAPK14	FOS	MAPK1	RELA	AKT1	IL6		
BP	Cellular response to chemical stress	FOS	MAPK1	JUN	RELA	AKT1	IL6		
BP	Response to reactive oxygen species	FOS	MAPK1	JUN	RELA	STAT1	AKT1	IL6	
BP	Response to oxidative stress	FOS	MAPK1	JUN	RELA	STAT1	AKT1	IL6	
BP	Cellular response to oxidative stress	FOS	MAPK1	JUN	RELA	AKT1	IL6		
BP	Reactive oxygen species metabolic process	MAPK14	MAPK1	AKT1					
BP	Cellular response to biotic stimulus	MAPK14	MAPK1	RELA	AKT1	IL6			
BP	Response to nutrient levels	MAPK1	JUN	RELA	STAT1	AKT1			
BP	Cellular response to lipopolysaccharide	MAPK14	MAPK1	RELA	AKT1	IL6			
CC	Membrane raft	MAPK1							
CC	Membrane microdomain	MAPK1							
CC	Membrane region	MAPK1							
CC	Caveola	MAPK1							
CC	Plasma membrane raft	MAPK1							
CC	Vesicle lumen	MAPK14	MAPK1						
CC	Secretory granule lumen	MAPK14	MAPK1						
CC	Cytoplasmic vesicle lumen	MAPK14	MAPK1						
CC	Integral component of the presynaptic membrane								
CC	Intrinsic component of the presynaptic membrane								
MF	Cytokine receptor binding	IL6							
MF	DNA-binding transcription factor binding	MAPK14	FOS	MAPK1	JUN	MYC	RELA	ESR1	STAT1
MF	Cytokine activity	STAT1	IL6						
MF	RNA polymerase II-specific DNA-binding transcription factor binding	MAPK14	FOS	MAPK1	JUN	RELA	ESR1		
MF	Signaling receptor activator activity	STAT1	IL6						
MF	Receptor ligand activity	IL6							
MF	Repressing transcription factor binding	MYC	RELA	STAT1					
MF	Integrin binding								
MF	Ubiquitin-like protein ligase binding	JUN	RELA	STAT1					
MF	Growth factor receptor binding	IL6							
KEGG	AGE-RAGE signaling pathway in diabetic complications	MAPK14	MAPK1	JUN	RELA	STAT1	AKT1		
KEGG	Lipid and atherosclerosis	MAPK14	FOS	MAPK1	JUN	RELA	AKT1		
KEGG	Fluid shear stress and atherosclerosis	MAPK14	FOS	MAPK1	JUN	RELA	AKT1		
KEGG	IL-17 signaling pathway	MAPK14	FOS	MAPK1	JUN	RELA	AKT1		
KEGG	TNF signaling pathway	MAPK14	FOS	MAPK1	JUN	RELA	AKT1		
KEGG	Kaposi sarcoma-associated herpesvirus infection	MAPK14	FOS	MAPK1	JUN	MYC	RELA	STAT1	
KEGG	Hepatitis B	MAPK14	FOS	MAPK1	JUN	MYC	RELA	STAT1	AKT1
KEGG	HIF-1 signaling pathway	MAPK1	RELA	AKT1					
KEGG	Human cytomegalovirus infection	MAPK14	MAPK1	MYC	RELA	AKT1			
KEGG	Chagas disease	MAPK14	FOS	MAPK1	JUN	RELA	AKT1		

### Zea-Longa Scores

**FIGURE 7:**
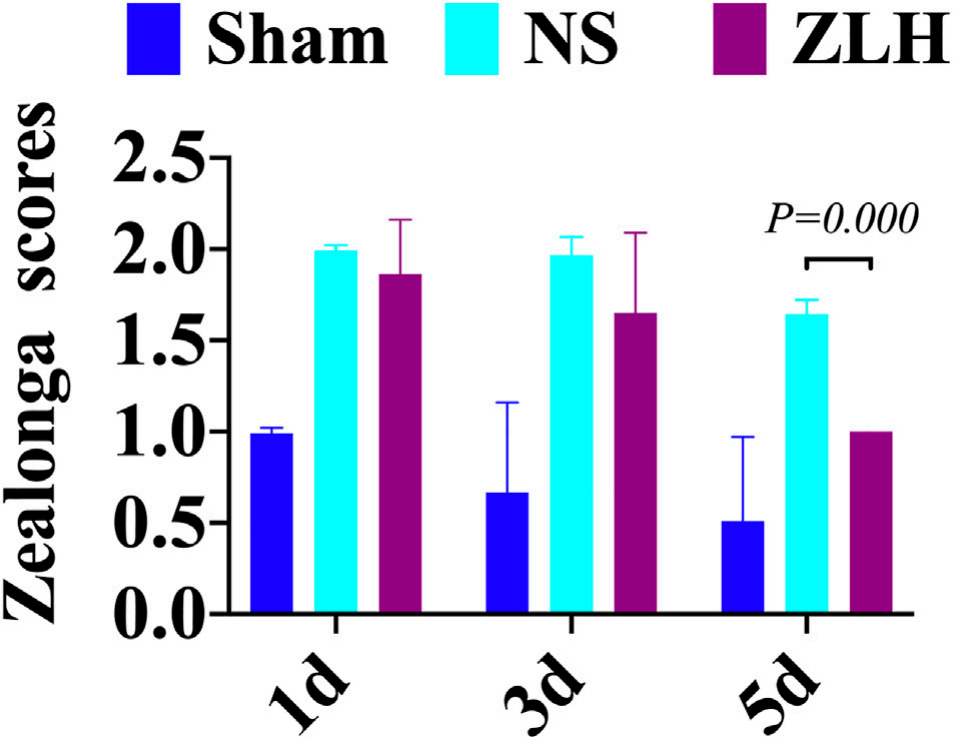
Zea-Longa scores at 1d, 3d, and 5d after modeling in the sham, NS, and ZLH groups.

Zea-Longa scores were performed on the sham group, NS group, and ZLH group at 1, 3, and 5 days after surgery. The results showed a significant increase in neurological dysfunction in the NS group compared to the sham group at 5 days after surgery, whereas it reversed in the ZLH-treated group, compared to the NS group (*p* = 0.000, Figure 7).

## Discussion

In the present study, we confirmed the effect of ZLH on neurological function repair in brain ischemic rats and determined the gene network in the cerebral ischemia–reperfusion model (Sun et al., 2015). We screened the relevant components and targets of ZLH in TCMSP and found the cerebral ischemia–reperfusion genes in GeneCards (Huang et al., 2020; Li et al., 2021) and then conducted a Venny intersection diagram to show cross genes, in which, 126 genes were selected in the intersection between ZLH and cerebral ischemia–reperfusion. The GO enrichment and KEGG signaling pathways are linked to lipopolysaccharide, membrane raft, cytokine receptor binding, and AGE-RAGE signaling pathway in diabetic complications, respectively (Ding and Zhang, 2017). Then, the top 10 gene pairs with the most close relationship in PPI network analysis reported were FLT1: VEGFA, AKT1: NOS3, CAV1: EGFR, CAV1: NOS3, CCND1: CDKN1A, CCND1: ESR1, CHUK: NFKBIA, EGF: EGFR, ESR1: JUN, and FOS: JUN, whereas the top 10 hub genes were screened out according to the degree value (Martino et al., 2021). Moreover, molecular docking confirmed that ZLH could form a stable molecular binding pattern, which targets directly with MAPK14, AKT1, FOS, IL6, MPK1 MYC, ESR1, JUN, RELA, and STAT1, but not with ESR1–Guizhi and Rela–Guizhi (Pinzi and Rastelli, 2019). Our results confirmed that multiple genes regulate and interact in ischemia–reperfusion cerebrum after ZLH treatment (Cui et al., 2020), which added new evidence to explain the mechanism of ZLH improving neurological function in cerebral ischemia–reperfusion rats.

### Protective Effect of ZLH in Brain Ischemic Rats

After the brain ischemia model was successfully established and the high-dose treatment of ZLH was used, we found that the score of neurological impairment was higher in brain ischemia, and it is lower in the ZLH group at 5 days. This suggested that the neurological function of cerebral ischemia–reperfusion rats was remarkably improved after high-dose administration of ZLH. Previously, it has been reported that ZLH exhibited a positive effect for the treatment of brain damage (Han et al., 2014), but the protective mechanism of ZLH keeps to be known.

### Pharmacological Analysis of Network and Its Significance

Network pharmacology can explain the relationship between drugs and gene network of diseases. Through network analysis, different genes regulating the disease and interacting genes on diseases could be found. Network pharmacology also analyzes the mechanism of action of TCM prescriptions on disease, therefore providing a new way to explain the molecular mechanism of TCM efficacy (Liu et al., 2020). Despite ZLH being used to dispel wind and dehumidify, relieve pain, and treat vascular headache and migraine, the molecular mechanism is largely unknown (She et al., 2019). Previously, it was only reported that ZLH has a therapeutic effect on acute cerebral infarction, and its safety and effectiveness were systematically evaluated (Liu et al., 2021); however, the gene network of ZLH is waiting to be explored. In this study, the gene network analysis and molecular docking between ZLH and cerebral ischemia disease were conducted (Liang et al., 2021), which laid the foundation for studying the mechanism of ZLH in treating cerebral ischemia–reperfusion.

### GO Enrichment and KEGG Signaling Pathway Analysis Were Performed on the Core Intersection Genes

The genes of ZLH were intersected with the genes of cerebral ischemia–reperfusion, and 126 intersection genes were obtained. The GO enrichment and KEGG signaling pathway analysis of the intersection genes showed that these genes were located in lipopolysaccharide as the most important BP. The main component of lipopolysaccharide is the outer membrane of Gram-negative bacteria, which causes immune response and plays an early warning role in the inflammatory reaction after cerebral ischemia–reperfusion (Park and Lee, 2013). The most important CC includes membrane raft, which is produced by the interaction between lipids (Quinn, 2010) and plays a regulatory role in the area around cerebral infarction (Ruscher et al., 2011). The most important MF was cytokine receptor binding, which activates intracellular enzymes and thus regulates gene expression (Spangler et al., 2015). The AGE-RAGE signaling pathway in diabetic complications is the top one signaling pathway, which indicates that patients with cerebral ischemia may develop hyperglycemia, and AGE-RAGE has harmful effects on neuron injury and inflammation in diabetic patients (Weil, 2012). The second pathway, lipid and atherosclerosis, is associated with the formation of atherosclerotic plaques (Bäck, 2009). Fluid shear stress and atherosclerosis as the third pathway plays an important role in atherosclerotic plaque vulnerability (Chen et al., 2019). The fourth pathway, IL-17 signaling pathway, is an effective pro-inflammatory cytokine that plays a role in inflammatory response after cerebral infarction (Liu Ting et al., 2019). Moreover, the TNF signaling pathway, the fifth signaling pathway, retracted inflammatory response after cerebral ischemia–reperfusion injury (Patel et al., 2014). The sixth pathway, Kaposi sarcoma-associated herpesvirus infection, which is the pathogen of malignant tumors, should be protected from viral infection after defective reperfusion (Fröhlich and Grundhoff, 2020). The seventh pathway, hepatitis B, or hepatitis virus, and the eighth pathway, HIF-1 signaling pathway, may play an important regulatory role in reducing oxidative stress response and inflammatory response after stroke (Zhang et al., 2021). The ninth pathway involved is human cytomegalovirus infection that may occur after cerebral ischemia–reperfusion. Lastly, the tenth pathway, Chagas disease, can lead to chronic diseases such as stroke when the acute infection subsides (Pérez-Molina and Molina, 2018). The summary of these pathways provided the crucial evidence to understand the molecular events in brain ischemic rats after ZLH treatment.

### PPI Interaction and Hub Gene Screening

The top 10 genes with the closest relationship were obtained, and we found that CCND1, ESR1, and JUN were connected with multiple genes in the top 10 pairs (Chakraborty et al., 2014). These closely related genes may play an important role in the treatment of cerebral ischemia–reperfusion with ZLH. The top 10 hub genes screened by the degree value in Cytoscape may be involved in a variety of cellular processes as the core regulatory genes in drugs and diseases (Deng et al., 2019).

### Molecular Docking Validation

As key nodes, the 10 hub genes were speculated as core genes in cerebral ischemia–reperfusion with ZLH treatment (Wei et al., 2020). Each of these 10 genes is linked to the corresponding drug ligand, which was revealed by molecular docking (Xia et al., 2020). From molecular docking analysis, it was found that *Astragalus* was docked with MAPK14, AKT1, FOS, IL6, MPK1 MYC, ESR1, JUN, RELA, and STAT1; Dahuang was directly docked with JUN, ESR1, and MYC; Cassia twig was docked with JUN, ESR1, and RELA. However, ESR1 and RELA had no direct interaction with Cassia twig. Therefore, through molecular docking, we provided the vital explanation for the drug directly targeting the molecule (Jiao et al., 2021).

### Network Relationships Between the Drugs and Diseases

By constructing a disease–drug active component–key target–KEGG pathway map, it can find the network interactions between the related targets (Niu et al., 2021). In this study, it was shown that the MOL number of ZLH had a relatively close node connection with cerebral ischemia–reperfusion and KEGG signaling pathways, which illustrates the interaction between ZLH and cerebral ischemia–reperfusion (He et al., 2020).

### The Functional Implication of Hub Genes From PPI in GO and KEGG Pathway

The relation of hub genes with PPI, GO, and KEGG pathway was analyzed. Several important genes were linked with the different cellular locations, biological processes, and molecular functions, meanwhile sharing the different signal pathways (Xie et al., 2018). MAPK4, Jun, Fos, Real, and IL-6 were found located in four BPs and seven signal pathways, which means that some hub genes were scattered in different pathways. In turn, the same signal pathway may include different genes, simultaneously. These showed that hub molecules from PPI take part in the core biological process or signal transduction in our observation. To our knowledge, this is the first time to reveal the relation among PPI, GO, and KEGG in brain ischemic rats subjected to ZLH treatment.

## Conclusion

In summary, we confirmed the effect of ZLH in protecting the ischemic brain from injury, and by using network pharmacology, molecular docking, and bioinformatics analysis, we explained the net mechanism in cerebral ischemia–reperfusion treated with ZLH. The results revealed that the effect of ZLH in improving neurological function is associated with multiple gene expressions in cerebral ischemia–reperfusion rats. Despite determining the effect of ZLH in treating brain ischemia and explaining the associated network mechanism, we suggest that the follow-up experimental research is absolutely needed to be developed to validate the deep mechanism.

## Data Availability

The original contributions presented in the study are included in the article/Supplementary Material; further inquiries can be directed to the corresponding authors.
